# Age-specific percentile-based reference curve of serum procalcitonin concentrations in Japanese preterm infants

**DOI:** 10.1038/srep23871

**Published:** 2016-04-01

**Authors:** Noriko Fukuzumi, Kayo Osawa, Itsuko Sato, Sota Iwatani, Ruri Ishino, Nobuhide Hayashi, Kazumoto Iijima, Jun Saegusa, Ichiro Morioka

**Affiliations:** 1 Department of Clinical Laboratory, Kobe University Hospital, Kobe, 6500017, Japan; 2 Department of Biophysics, Kobe University Graduate School of Health Sciences, Kobe, 6540142, Japan; 3 Department of Infection Control and Prevention, Kobe University Hospital, Kobe, 6500017, Japan; 4 Department of Pediatrics, Kobe University Graduate School of Medicine, Kobe, 6500017, Japan

## Abstract

Procalcitonin (PCT) levels are elevated early after birth in newborn infants; however, the physiological features and reference of serum PCT concentrations have not been fully studied in preterm infants. The aims of the current study were to establish an age-specific percentile-based reference curve of serum PCT concentrations in preterm infants and determine the features. The PCT concentration peaked in infants at 1 day old and decreased thereafter. At 1 day old, serum PCT concentrations in preterm infants <34 weeks’ gestational age were higher than those in late preterm infants between 34 and 36 weeks’ gestational age or term infants ≥37 weeks’ gestational age. Although the 50-percentile value in late preterm and term infants reached the adult normal level (0.1 ng/mL) at 5 days old, it did not in preterm infants. It took 9 weeks for preterm infants to reach it. Serum PCT concentrations at onset in late-onset infected preterm infants were over the 95-percentile value. We showed that the physiological feature in preterm infants was significantly different from that in late preterm infants, even in those <37 weeks’ gestational age. To detect late-onset bacterial infection and sepsis, an age-specific percentile-based reference curve may be useful in preterm infants.

Procalcitonin (PCT), a prohormone of calcitonin, is composed of 116 amino acids, and is produced from thyroid C cells^1^,^2^. Elevation of the serum PCT concentration is a more sensitive marker than the white blood cell (WBC) count and C-reactive protein (CRP) level in the early identification of bacterial infection and sepsis in adults and children^3^,^4^,^5^,^6^,^7^,^8^,^9^,^10^, because PCT is released into the blood several hours after infection onset^1^,^2^,^11^,^12^. The reference range of serum PCT levels in adults and children is less than 0.1 ng/mL^3^,^13^, and the cut-off value for the diagnosis of bacterial infections or sepsis has been established at 0.5 ng/mL^4^,^5^,^6^,^7^,^8^,^9^. In newborns, however, as PCT levels are known to be elevated early after birth^14^,^15^,^16^,^17^,^18^,^19^,^20^,^21^, the serum PCT value must be carefully interpreted when judging infection, and it is necessary to set a different reference for children.

In some main early studies, Chiesa *et al*., for the first time, showed an age-specific reference from birth to 48 hours for the serum PCT in term infants with a peak around 20 ng/mL (the 95-percentile value) at 24 hours after birth, which decreased thereafter^15^. Their follow-up study and Turner *et al*.’s study reported that physiological changes in serum PCT levels early after birth are different between preterm and term infants^16^,^18^. Based on such evidence, some researchers have set cut-off values corresponding to the time after birth, and they have shown that serum PCT was a marker with a high accuracy for diagnosing neonatal sepsis or bacterial infection in newborns^15^,^16^,^19^,^22^,^23^,^24^.

Among newborn infants born <37 weeks’ gestational age (GA), late preterm and preterm infants born at 34–36 and <34 weeks’ GA, respectively, have a different mature status of immune, endocrine, or metabolism systems^25^. Therefore, serum PCT concentrations of infants should be assessed using distinguished references for each infant population. However, the features of physiological changes and reference in these infant populations have not been fully studied. Furthermore, because preterm infants hospitalized in the neonatal intensive care unit (NICU) for a long period are at a very high risk of health care-associated late-onset sepsis or bacterial infection^26^, their long-term normal reference range of serum PCT should be established.

The aims of the present study were to 1) establish age-specific percentile-based reference curves of serum PCT in late preterm and preterm infants early after birth and determine the physiological features of PCT; 2) determine the age when infants’ serum PCT reaches ≤0.1 ng/mL, which is the reference range for adults and children, in preterm infants; and 3) evaluate changes in serum PCT concentrations in preterm infants who develop late-onset sepsis or bacterial infection using the 95-percentile reference curve we established.

## Results

### Patients’ characteristics

Patients’ characteristics by each infant group are summarized in Table 1. The birth weight decreased as the GA decreased, which is what we expected. The body size at birth, sex, delivery mode, and Apgar scores had different proportions among the infant groups.

### Serum PCT concentrations from birth to 5 days old

**Figure 1:**
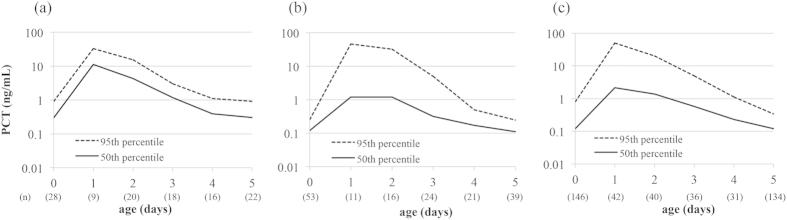
Postnatal age-specific percentile-based curves of serum PCT concentrations in the first 5 days of age. (**a**) Preterm, (**b**) late preterm, and (**c**) term infants from birth to 5 days old. PCT, procalcitonin.

Figure 1 shows the postnatal age-specific percentile-based reference curves of serum PCT concentrations in preterm, late preterm, and term infants from birth to 5 days old. The PCT concentration peaked at 1 day old and decreased thereafter in all infant groups. At 1 day old, the serum PCT concentration in preterm infants was higher compared with that in late preterm or term infants (50-percentile values were 11.1 ng/mL in preterm, 1.2 ng/mL in late preterm, and 2.2 ng/mL in term infants). In contrast, the 50-percentile value of late preterm and term infants reached about 0.1 ng/mL at 5 days old (0.11 ng/mL and 0.12 ng/mL in late preterm and preterm infants, respectively), but not in preterm infants (0.31 ng/mL).

### Comparison of serum PCT concentrations among the groups

**Figure 2:**
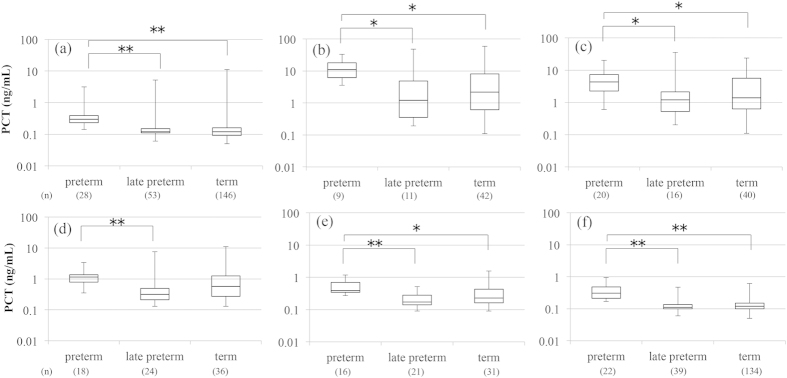
Comparison of serum PCT concentrations among preterm, late preterm, and term infants. (**a**) At birth, (**b**) 1 day old, (**c**) 2 days old, (**d**) 3 days old, (**e**) 4 days old, and (**f**) 5 days old. Data were given as median and range (25th percentile to 75th percentile). *p < 0.05, **p < 0.01. PCT, procalcitonin.

Serum PCT concentrations were compared among the preterm, late preterm, and term infant groups each day of age (Fig. 2). Serum PCT concentrations in preterm infants were significantly higher than those in late preterm and term infants from birth to 5 days old, except for the values between preterm and term infants at 3 days old. There were no significant differences in serum PCT concentrations between late preterm and term infants from birth to 5 days old.

### Serum PCT concentrations until 12 weeks old in preterm infants

**Figure 3:**
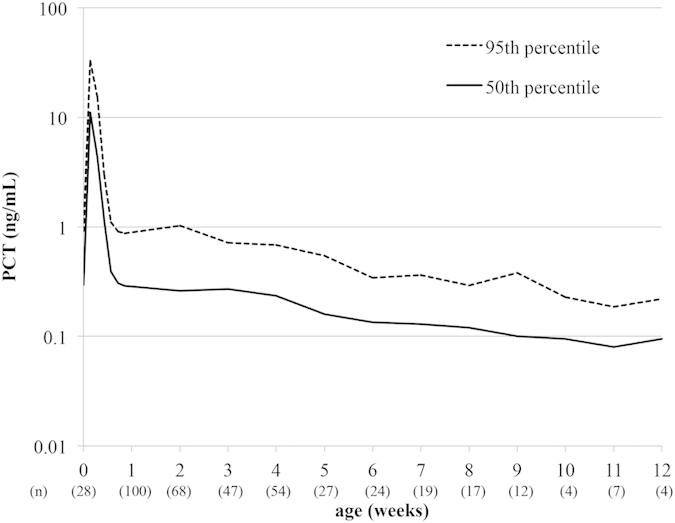
Postnatal age-specific percentile curve of serum PCT concentrations from birth to 12 weeks old in preterm infants. PCT, procalcitonin.

The time course of serum PCT concentrations from birth to 12 weeks old in preterm infants is shown in Fig. 3. The 50-percentile value of the serum PCT concentration reached adult normal levels (0.1 ng/mL) after 9 weeks old.

### Late-onset infected cases

#### *Case* 1

A Japanese infant weighing 482 g was born vaginally at 22 weeks and 4 days. At 42 days old, dyschromia and hypotension appeared, and the blood test showed that the WBC count was 16,900/μL (stab cells: 28%, segmented cells: 49%), platelet count was 0.3 × 10^4^/μL, and serum CRP value was 13.64 mg/dL. The infant was treated with cefotaxime and vancomycin. *Enterobacter cloacae* was detected from the blood culture. This case was diagnosed with culture-proven sepsis.

#### *Case* 2

A Japanese infant weighing 1,014 g was born by emergency caesarean section at 27 weeks and 5 days. At 12 days old, the infant was extubated; however, an apnea attack occurred. Chest radiography showed infiltration in the lung field, and the blood test showed that the WBC count was 32,500/μL (neutrophils: 76%) and the serum CRP value was 1.75 mg/dL. The infant was treated with cefmetazole. *Pseudomonas aeruginosa* was detected from the tracheal tube culture. This patient was diagnosed with ventilator-associated pneumonia.

Case 3. 

A Japanese infant weighing 1,546 g was born by emergency caesarean section at 30 weeks and 3 days. At 55 days old, an apnea attack occurred. The blood test showed that the WBC count was 9,600/μL (neutrophils: 52%) and serum CRP value was 3.73 mg/dL. The infant was treated with tazobactam/piperacillin and arbekacin. As no bacteria were detected from the blood culture, this patient was diagnosed with clinical sepsis.

### Time course of serum PCT concentrations of late-onset infected preterm infants

**Figure 4:**
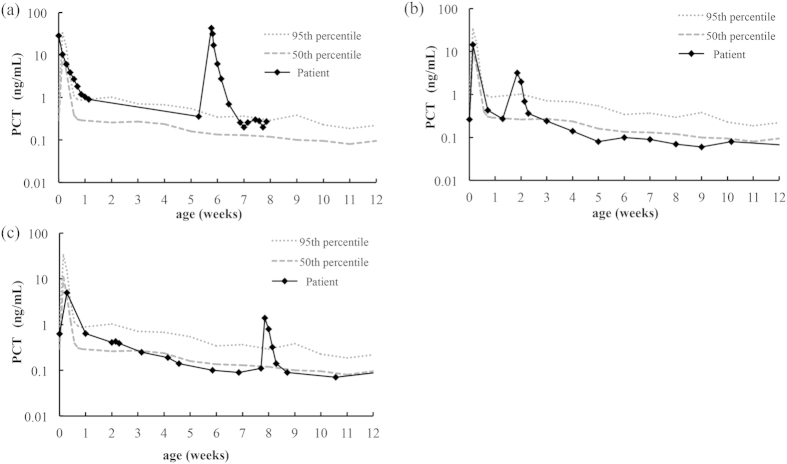
Time course of serum PCT concentrations in three various late-onset infected cases of preterm infants. **(a**) Case 1, culture-proven sepsis with *Enterobacter cloacae* at 42 days old; (**b**) Case 2, ventilator-associated pneumonia with *Pseudomonas aeruginosa* at 12 days old; and (**c**) Case 3, clinical sepsis at 55 days old.

Figure 4 shows the changes in serum PCT concentrations in late-onset infected preterm infants. All cases clearly showed a higher value at onset than the 95-percentile value, and this returned within the reference range, indicating improvement.

## Discussion

In the current report, we established a postnatal age-specific percentile-based reference of serum PCT in newborn infants, and showed that physiological changes in late preterm infants were significantly different from those in preterm infants, but not in term infants. Furthermore, we established a postnatal age-specific percentile-based reference in preterm infants, and determined that the serum PCT concentration reaches adult normal levels (0.1 ng/mL) after 9 weeks old. As the serum PCT concentration in preterm infants is age-dependent, the reference for the serum PCT concentration should be used before 9 weeks of age. Using this reference curve, preterm infants with late-onset sepsis or a bacterial infection were clearly over the 95-percentile value for PCT at the time of onset.

Neonatal sepsis or bacterial infection is a major cause of growth outcomes and neurological sequelae, especially in preterm infants^27^. As an early diagnosis and subsequent antibiotic therapy are needed to improve infants’ outcomes, neonatologists are eagerly awaiting the clinical application of a new early diagnostic marker because existing markers such as the CRP level have a low sensitivity in preterm infants^28^. Although earlier studies have reported that PCT was a more sensitive marker than existing markers such as the WBC count, CRP level, and interleukin-6 levels in adults and children in the clinical setting^5^,^6^,^7^,^8^,^9^,^10^, the sensitivity and specificity of serum PCT in newborns were low compared to those in adults for diagnosing sepsis (respective sensitivity and specificity: 80% and 94% in adults^29^ and 77% and 62% in newborns^30^ using a cut-off value of 1 ng/mL). The reason for this low accuracy may be why serum PCT becomes physiologically elevated after birth even in non-infected newborns (e.g., those with respiratory and hemodynamic disorders or asphyxia even with mild symptoms)^31^,^32^,^33^,^34^. Therefore, to use serum PCT in the neonatal clinical setting, a nomogram and reference curve of the serum PCT concentration are needed.

Some studies in western countries have already reported the nomogram and references of the serum PCT concentration within 5 days after birth^15^,^16^,^18^. This is the first report of a serum PCT reference curve in a Japanese preterm infant population. Concordant with previous studies^15^,^16^,^18^, our results also observed that the serum PCT concentration peaked at 1 day old, decreased thereafter, and returned to adult normal levels until 5 days old in term and late preterm infants, suggesting no ethnic difference. Physiological changes of the serum PCT in late preterm infants should be similar to that in term infants. However, the PCT concentration in preterm infants reached adult or child normal levels after 9 weeks, and the duration was longer than that in late preterm and term infants (5 days old in this study), and in preterm infants studied previously (96–120 hours)^16^,^18^. Similar to our results, Hahn *et al*. reported the reference interval of serum PCT from 7–60 days postnatally in preterm infants, and preterm infants ≤32 weeks’ GA still had age dependency until 60 days old^35^. Preterm infants may take a long time to reach adult normal levels due to 5- to 10-fold higher PCT amounts at peak (1 day old) and more immature endocrine and metabolism systems than term infants (generally a half-life of PCT is around 22–26 hours^1^). The serum PCT concentration in preterm infants is higher compared with that in term infants, because PCT, a precursor of calcitonin, may be associated with osteogenesis and the metabolism of calcium^2^. Further studies are needed to determine the mechanism of this phenomenon.

Our reference curve only used the upper limit of the 95 percentile. The reason for this was that PCT has a non-detection level or an extremely low level in the blood of healthy conditions except for newborn infants because it is produced from the thyroid and transformed into calcitonin quickly^2^. When adopting this percentile curve in preterm infants to various late-onset infected cases, the serum PCT was over the 95-percentile value at onset in all cases. This postnatal age-specific 95-percentile value or other percentile values such as the 90 percentile or 97.5 percentile may be useful for detecting late-onset sepsis or bacterial infection in preterm infants.

Limitations of this study are as follows: 1) as this was a retrospective study, the sample number was biased at each time point after birth. Ideally, the same infants should be longitudinally sampled at every time point to establish the reference; however, the necessary blood amount for measuring the PCT concentration is relatively too much in newborn infants; therefore, this would be unethical. The usefulness of this 95-percentile postnatal age-specific nomogram in preterm infants was not fully determined because there were only three late-onset infected infants. To draw this conclusion, further studies with more late-onset infected cases should be performed, and the test performance characteristics should be shown using the determined cut-off criteria.

In conclusion, we established a reference for serum PCT concentrations in Japanese preterm, late preterm, and term infants during the early period after birth, and for the first time, we found that GA dependency was different at the boundary of 34 weeks’ GA. In addition, the percentile-based curve of the longer period until 12 weeks old was established in preterm infants, and we determined that there is age-dependency until 9 weeks old. An age-specific percentile-based reference curve may be useful for diagnosing late-onset bacterial infection and sepsis in preterm infants.

## Methods

### Study design and subjects

This retrospective study was conducted in Japanese newborns admitted to our NICU at Kobe University Hospital, Japan between June 2014 and December 2014. To establish an age-specific percentile-based reference curve, newborns were excluded if they met the following conditions: (1) congenital malformations and genetic disorders, including a chromosomal abnormality; (2) clinical signs of infection, including sepsis and treatment with antibiotics, or a positive culture; and (3) a history of surgery because this condition may affect the physiological PCT status. Other infants were enrolled in this study. To analyse changes in serum PCT concentrations in late-onset infection, 3 late-onset infected preterm infants were enrolled. Residual serum samples after completing routine laboratory tests were used for this study. Data collection and the use of human materials for this study were approved by the Ethical Committee of Kobe University Graduate School of Medicine (no. 1688), and written informed consent was obtained from the parents of all enrolled newborn infants. The methods were carried out in accordance with the approved guidelines.

During the study period, 299 newborn patients were admitted to our NICU. Of these, 16 patients were excluded because 4 were non-Japanese, 7 had congenital malformations and genetic disorders, 4 had infections (3 late-onset and 1 early onset), and 1 had undergone surgery. Finally, serum samples (n = 1,267) were obtained from 283 newborn patients at birth to 136 days old at the discretion of the attending neonatologist, and based on their clinical conditions, with multiple serum samples obtained from some patients at different days.

### Definition

The onset of infection within 72 hours and after 72 hours of birth was defined as early- and late-onset infection, respectively. Small-, appropriate-, or heavy-for-GA was defined as a birthweight less than 10th percentile, between 10th and 90th percentile, or more than 90th percentile for the same GA in a Japanese population^36^.

### Serum PCT measurement

Using 30 μL of serum, the serum PCT concentration was measured by electrochemical luminescence immunoassay using the COBAS 8000e analyser (Roche Diagnostics, Basel, Switzerland). Data are shown as ng/mL.

### Study methods

The enrolled newborns were divided into three infant groups as follows: preterm (<34 weeks’ GA, n = 37), late preterm (34–36 weeks’ GA, n = 61), and term infants (≥37 weeks’ GA, n = 185). First, a postnatal age-specific percentile-based reference curve was established for serum PCT from birth to 5 days old in preterm, late preterm, and term infants. Second, serum PCT concentrations were compared each day among infants within 5 days of age. Third, a postnatal age-specific percentile-based reference curve of serum PCT concentrations was created until the concentrations returned to adult normal levels (0.1 ng/mL) in preterm infants. Fourth, the time course in serum PCT concentrations in late-onset infected infants were analysed using our age-specific percentile-based reference curve for preterm infants.

### Statistical analysis

The Kruskal-Wallis test was used to compare multiple independent data sets, and differences between the individual data sets were assessed using the all-pairwise test from the Steel-Dwass method. The percentile was determined by the non-parametric method. Statistically significant differences were considered for p values <0.05.

## Additional Information

**How to cite this article**: Fukuzumi, N. *et al*. Age-specific percentile-based reference curve of serum procalcitonin concentrations in Japanese preterm infants. *Sci. Rep.* **6**, 23871; doi: 10.1038/srep23871 (2016).

## Figures and Tables

**Table 1:** Characteristics of enrolled infants.

	All infants N = 283	Preterm (<34 weeks’ GA) n = 37	Late preterm (34–36 weeks’ GA) n = 61	Term (≥37 weeks’ GA) n = 185
GA, weeks	37 (22–41)	30 (22–33)	36 (34–36)	38 (37–41)
Birth weight, g	2,698 (338–4,176)	1,270 (338–2,244)	2,350 (1,368–3,460)	2,956 (2,098–4,176)
Body size at birth				
AGA	228 (80)	29 (78)	46 (75)	153 (83)
SGA	33 (12)	8 (22)	12 (20)	13 (7)
HGA	22 (8)	0 (0)	3 (5)	19 (10)
Male sex	188 (66)	29 (78)	48 (79)	111 (60)
Delivery mode				
Vaginal delivery	143 (51)	11 (30)	22 (36)	110 (59)
Caesarean section	140 (49)	26 (70)	39 (64)	75 (41)
Apgar score (1 min)				
≤7	44 (16)	19 (51)	7 (11)	18 (10)
≤3	9 (3)	6 (16)	1 (2)	2 (1)

Data are shown as median (range) or number (%). AGA, appropriate-for-gestational age; GA, gestational age; HGA, heavy-for-gestational age; SGA, small-for-gestational age.
